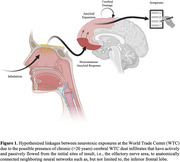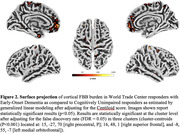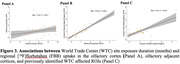# Exposure Severity and Cognitive Dysfunction are Mediated by Low‐Grade Cerebral Amyloidosis: A PET/MRI study of World Trade Center responders

**DOI:** 10.1002/alz.093801

**Published:** 2025-01-09

**Authors:** Minos Kritikos, Juin W. Zhou, Chuan Huang, Paul Vaska, Sean A.P. Clouston, Benjamin J Luft

**Affiliations:** ^1^ Stony Brook University, Stony Brook, NY USA; ^2^ Stony Brook University Hospital, Stony Brook, NY USA

## Abstract

**Background:**

World Trade Center (WTC) responders endured exposures to neurotoxic dust particulate matter. This neuroimaging study examined the presence of amyloidosis in Alzheimer’s disease (AD) regions of interest (ROIs) and associations with exposure duration.

**Method:**

Simultaneous positron‐emission tomography with [18F]‐florbetaben and magnetic resonance neuroimaging was acquired on 34 middle aged WTC responders. Centiloid scale used mean standardized uptake value ratio (SUVR) with between subjects comparisons of cognitively unimpaired (n = 17) and early‐onset dementia (n = 17) responders. Pathway analyses investigated potential neuropathological cascades.

**Result:**

Only one subject was Centiloid positive. However, considerable SUVRs in non‐AD ROIs associated with changes in cortical mean diffusivity, cortical thickness, cognition, and exposure duration, mediated by the olfactory bulb.

**Conclusion:**

Fibrillar amyloidosis may have originated in, and spread from the olfactory cortex because of prolonged exposures to WTC neurotoxic dust. Our study also identifies a non‐AD neuropathological topology of amyloidosis, which associated with lower measures of brain health.